# A seven-year experience with patients with juvenile nasopharyngeal angiofibroma

**DOI:** 10.1590/S1808-86942010000200016

**Published:** 2015-10-19

**Authors:** Alfredo Lara Gaillard, Vanessa Menegatti Anastácio, Vânia Belintani Piatto, José Victor Maniglia, Fernando Drimel Molina

**Affiliations:** Third year medical resident in Otorhinolaryngology and Head & Neck Surgery; Third year medical resident in Otorhinolaryngology and Head & Neck Surgery; Doctorate in health sciences, adjunct professor in the Otorhinolaryngology and Head & Neck Surgery Department - FAMERP; Chair professor, adjunct professor in the Otorhinolaryngology and Head & Neck Surgery Department; Doctor, head of the Otorhinolaryngology and Head & Neck Surgery Department outpatient unit

**Keywords:** angiofibroma, treatment, embolization, surgery, epistaxis

## Abstract

Juvenile nasopharyngeal angiofibroma (JNA) is a rare tumor in adolescent males. It originates in the nasopharynx.

**Aim:**

to present the experience of JNA management at an Otorhinolaryngology Service between 2001 and 2008.

**Materials and Methods:**

Demographical data, clinical presentation, investigations as well as the treatment of sixteen JNA patients were reviewed and collected from medical records from the ORL Service.

**Design:**

Cross-sectional, retrospective and descriptive study. Results: All JNA patients were male. The average age at diagnosis was 16.8 years (range 9–23 years). More than 56% of the patients were classified as Fisch II. Preoperative embolization was carried out in ten (62.5%) patients. All 16 patients were submitted to primary surgical resection. Two patients (66.7%) who didn't receive preoperative embolization required intraoperative blood transfusion. The overall recurrence rate was 43.75% and the cure rate was 93.75%.

**Conclusion:**

Preoperative embolization minimizes intraoperative blood loss. The recurrence rate was related to advanced tumoral stage at diagnostic and the lack of preoperative embolization. Surgery combined with preoperative embolization is the major treatment for JNA. All the patients should undergo preoperative imaging studies, especially CT, to assist in surgical planning and follow-up.

## INTRODUCTION

The juvenile nasopharyngeal angiofibroma is a rare benign vascular tumor representing about 0.5% of all head and neck neoplasms.^1^ Its incidence is 1:150,000, and it affects mostly male teenagers and young adults from ages 14 to 25 years.^2^ A few cases have been reported in men over 25 years and in female teenagers.^3^, ^4^, ^5^

It is thought that the anatomical origin of this tumor is the postero/lateral wall of the nasal cavity, close to the superior border of the sphenopalatine foramen. This tumor starts to grow on the submucosa of the floor of the nasopharynx, reaching the nasal septum and the posterior space of the nose, thereby causing a mass effect that may lead to airway obstruction. As the process is continuous, the anterior face of the sphenoidal sinus is destroyed and invaded. The tumor may grow towards the nasal fossa and extend to the posterior portion of the middle turbinate, which becomes a common part of the tumor mass. The angiofibroma may extend laterally towards the pterygomaxillary fossa and destroy the posterior wall of the maxillary sinus. Eventually, the tumor may invade the intratemporal fossa and the middle cranial fossa. This is a general view of the growth of this tumor. The actual modes of invasion into local structures may be unpredictable and far from a typical pattern.^6^^,^^7^

The macroscopic aspect of this tumor is a rounded, circumscribed, non-capsulated, mucosa-covered mass. The color depends on the vascular component; it may be pink-and-wine-colored or pale-whitish. The maxillary artery supplies most of the blood to these tumors, with secondary contributions from the ipsilateral internal carotid artery or the contralateral external carotid system. The histological aspect of these tumors shows fibrous connective tissue and vessels. The periphery of the tumor is edematous and infiltrated, with necrosis and capillary vessels. The deeper vessels are larger and multiply anastomotic, similar to a cavernous hemangioma. The hemorrhagic feature of this tumor is due to a single endothelial cell layer with no smooth muscle and a rigid fibrous stroma.^8^, ^9^, ^10^

The site and extension of tumor invasion should be defined; not recognizing the anatomical site may result in a high rate of incomplete resections, which may already be high because identifying the tumor capsule after prior embolization is difficult. Several staging systems have been proposed to make it possible to recognize the anatomical site of tumor invasion; Fisch's classification^11^ is currently used most often. According to this classification, the angiofibroma is classified as Type I when the tumor is restricted to the nasal cavity and the nasopharynx without bone destruction; Type II when the tumor invades the pterygomaxillary fossa and maxillary, sphenoidal and ethmoid sinuses with bone destruction; Type III when the tumor invades the intratemporal fossa, the orbit and the parasellar region, but remaining lateral to the cavernous sinus; and Type IV when the tumor invades the cavernous sinus, the optic chiasma, and the pituitary fossa.^11^

The classical clinical presentation of the angiofibroma is unilateral nasal block and/or epistaxis, rhinorrhea, and occasionally pain. Because of its invasive nature, the tumor may cause facial deformity, and proptosis, changes in visual acuity and cranial nerve palsy if it reaches the orbit and intracranial region.^12^^,^^13^

The diagnosis of angiofibroma is essentially based on a careful clinical history and a detailed physical examination to describe the signs and symptoms, the morphology and epidemiology, and the radiological findings. Imaging techniques have led to major advances in the diagnosis and treatment of angiofibromas. Nasal endoscopy, computed tomography, and magnetic resonance imaging help establish the site and extension of this tumor and the relations with blood vessels and nerves, all of which make it possible to precisely stage the tumor. Biopsies are not recommended because of the vascular and hemorrhagic nature of this tumor. Arteriography is done to assess the vascular supply in larger tumors and to make it possible to embolize these vascular lesions to reduce intraoperative bleeding.^14^, ^15^, ^16^

Several tumoral or non-tumoral diseases may cause nasal block and epistaxis, which may be taken for an angiofibroma. The following conditions should be taken into account in the differential diagnosis: inflammatory polyps, angiomatous polyps, nasopharyngeal cysts, nasopharyngeal malignancies, nasopharyngeal fibrosarcomas and fibromas, upper maxillary malignancies, nasal fossa stesioneuroblastoma, adenoid hypertrophy, cervical vertebra cordomas, and retropharyngeal ganglia tuberculosis.^16^

Several surgical approaches have been described for the treatment of angiofibromas; these include the transpalatine approach, lateral rhinotomy, and medio-facial degloving. In any of these techniques, the recommended treatment of choice is preoperative tumor embolization to decrease intraoperative bleeding when removing the angiofibroma.^15^, ^16^, ^17^, ^18^, ^19^, ^20^, ^21^, ^22^

This study reports the experience of an Otorhinolaryngology and Head & Neck Surgery Department in a school hospital from 2001 to 2008 with the diagnosis and treatment results of patients with juvenile nasopharyngeal angiofibroma.

## SERIES AND METHOD

The institutional review board of our institution approved this study (protocol no. 3644/2009), in accordance with the regulating guidelines for research on humans in Resolution 196/96, of the Ministry of Health.

A cross-sectional retrospective study was carried out using data from a review of the records of 16 patients diagnosed with angiofibromas, from 2001 to 2008. The informed consent form was not needed in this context.

The following data were gathered: gender, age at the time of diagnosis, signs and symptoms, diagnostic examination, surgical approach, need for transfusion, preoperative embolization, complications, follow-up exams, recurrences, and reoperation. The Fisch (Fisch, 1983) criteria were applied to classify the tumors.

Percentages (%) were calculated and their standard deviations (SD), which were expressed as percentages (SD%).

## RESULTS

From 2001 to 2008, 16 male patients (100%) underwent treatment for angiofibromas. The ages ranged from 9 to 23 years, with a mean 16.8 years (SDM= 0.885). One patient (6.25%, SD%=24.20) was a child (7 to 10 years), 6 patients (37.5%, SD%=19.76) were teenagers (11 to 16 years), and 9 patients (56.25%, SD%=16.53) were young adults (17 to 25 years) at the time of diagnosis.

The most frequent signs and/or symptoms were epistaxis (93.75%, SD%=6.25) and nasal block (75%, SD%=12.5), followed by hyposmia (12.5%, SD%=23.38), bulging of the face (6.25%, DP%=24.20), and decreased visual acuity (6.25%, SD%=24.20). No patient reported rhinorrhea.

All patients underwent nasal endoscopy and computed tomography. No biopsies were carried out in these patients.

Based on the tomographic findings and the Fisch classification,11 4 patients (25.0%, SD%=21.65) were stage I, 9 patients (56.25%, SD%=16.53) were stage II, 3 patients (18.75%, SD%=15.61) were stage III, and there were no stage IV patients (0%).

Ten patients (62.5%, SD%=15.30) underwent arteriography with superselective embolization of branches of the maxillary artery 48 hours prior to surgery, with no complications.

All patients underwent surgery, as follows: medial facial degloving + Denker in 9 patients (56.25%, SD%=16.53), medial facial degloving + Denker + nasal endoscopy in 2 patients (12.5%, SD%=23.38), transpalatine in 2 patients (12.5%, SD%=23.38), nasal endoscopy in 2 patients (12.5%, SD%=23.38), and the LeFort I osteotomy in 1 (6.25%, SD%=24.20).

Three patients (18.75%, SD%=22.53) required intraoperative blood transfusions, of which 2 patients (66.67%, SD%=33.33) had not done preoperative embolization.

Nasal packing was done in all patients at the end of surgery and removed 48 to 72 hours later.

Only 2 patients (12.5%, SD%=23.38) had postoperative complications, such as an oroantral leak following a transpalatine approach, and lower turbinate and septal synechiae after nasal endoscopy; both complications were corrected with no additional morbidity.

Tumors recurred in 1 of 4 stage I patients (25%, SD%=43.30), 3 of 9 stage II patients (33%, SD%=27.14), and 3 of 3 stage III patients (100%). Preoperative embolization had been carried out in 3 (43%, DP%=28.58) of these 7 patients, and not on the remaining 4 patients (57%, SD%=24.75). The recurrence rate was 43.75% (SD%=18.75). The time to relapse after surgery ranged from 1 month to 3 years; all 7 patients were reoperated. Of these, only 1 (14.3%, SD%=35.0) presented a palatine fissure as a complication of reoperation, which was also corrected.

Follow-up of 15 patients is being done quarterly, except for one patient who abandoned follow-up since 2006; these patients undergo computed tomography for monitoring purposes, and no recurrences have been found to date. The follow-up time has ranged from 1 to 6 years; the mean is 2.3 years (SDM= 1.77). Based on this, the cure rate is 93.75% (SD%=6.25).

Tables 1 and 2 show the data gathered from 16 patient files.

**Table 1:** Patient data in relation to age at diagnosis, the need for embolization, surgical approach, and postoperative complications.

Patient	Fisch 11 staging	Age at diagnosis	Preoperative embolization	Time between embolization and surgery	Surgical approach	Intraoperative transfusion	Postoperative complications
1	II	19y6m	Yes	48 hs	Nasal endoscopy	—	—
2	III	14y10m	Yes	48 hs	Medio-facial degloving + Denker	—	—
3	III	15y7m	Yes	48 hs	Medio-facial degloving + Denker	3U	
4	II	17y10m	Yes	48 hs	Medio-facial degloving + Denker	—	—
5	II	12y2m	Yes	48 hs	Medio-facial degloving + Denker	—	—
6	II	23y3m	Yes	48 hs	Medio-facial degloving + Denker	—	—
7	II	20y	Yes	48 hs	Medio-facial degloving + Denker	—	—
8	I	20y5m	Yes	48 hs	Medio-facial degloving + Denker	—	—
9	II	17y6m	Yes	48 hs	Medio-facial degloving + Denker + nasal endoscopy	—	—
10	II	15y7m	Yes	48 hs	Medio-facial degloving + Denker	—	—
11	III	17y4m	No	—	LeFort I osteotomy	3U	—
12	II	15y11m	No	—	Medio-facial degloving + Denker	6U	—
13	I	9y1m	No	—	Medio-facial degloving + Denker + nasal endoscopy	—	—
14	II	16y5m	No	—	Transpalatine	—	—
15	I	21y4m	No	—	Transpalatine	—	Oroantral leak
16	I	19y11m	No	—	Nasal endoscopy	—	Lower turbinate and nasal septum synechia

**Table 2:** Patient data in relation to procedures after recurrences and follow-up.

Patient	Fisch^11^ staging	Recurrence/time after 1st surgery	Diagnostic exam	Reoperation	Intraoperative transfusion	Postoperative complication	Follow-up/Exams	New recurrence until 2008
1	II	No	—	—	—	—	Quarterly/TC+ control	No
2	III	Yes/5 months	Nasofibroscopy RM*	Degloving MF+ + Nasal endoscopic review	—	—	Quarterly/TC+ control	No
3	III	Yes/2 months	TC+	Nasal endoscopic review			Quarterly/TC+ control	No
4	II	No	—	—	—	—	Quarterly/TC+ control	No
5	II	No	—	—	—	—	Quarterly/TC+ control	No
6	II	No	—	—	—	—	Quarterly/TC+ control	No
7	II	No	—	—	—	—	Quarterly/TC+ control	No
8	I	No	—	—	—	—	Quarterly/TC+ control	No
9	II	No	—	—	—	—	Quarterly/TC+ control	No
10	II	Yes/1 month	Nasofibroscopy	Nasal endoscopic review	—	—	Quarterly/TC+ control	No
11	III	Yes/10 months	TC+	Degloving MF++ Nasal endoscopic review	—	—	Quarterly/TC+ control	No
12	II	Yes/5 months	TC+	Degloving MF+	4U	Fissura palatina	Quarterly/TC+ control	No
13	I	Yes/3 years	Nasofibroscopy	Nasal endoscopic revision	—	—	Quarterly/TC+ control	No
14	II	Yes/2 years	TC+	LeFort I osteotomy	1U		Quarterly/TC+ control until 2006	No until 2006
15	I	No	—	—	—	—	Quarterly/TC+ control	No
16	I	No	—	—	—	—	Quarterly/TC+ control	No

* *RM-Magnetic Resonance, +TC-Computed Tomography, +Medio-facial degloving

## DISCUSSION

The juvenile nasopharyngeal angiofibroma is, as the name suggests, a disease of young men.^6^ In the present study, all patients were male, and the mean age at the time of diagnosis was 16.8 years, which is similar to other published studies.^13^, ^14^, ^15^^,^^20^^,^^23^ Genetic studies have demonstrated a close relation between these angiomas and androgen receptor expression, suggesting that this tumor is androgen-dependent. This could explain why the prevalence is higher in males.^24^, ^25^

In most cases, the clinical presentation of the angiofibroma comprises the triad nose block, epistaxis, and a nasopharyngeal mass, which was similar to our findings in the study and in previously published papers.^13^^,^^14^^,^^26^^,^^27^

The diagnosis of angiofibroma is given based on the clinical history, the physical examination, and nasal endoscopy; imaging studies, such as computed tomography, may add further information, as was done in the patients comprising this study. The correlation between preoperative staging based on computed tomography and the Fisch criteria^11^ was possible in the 16 cases of this study. Computed tomography makes it possible to stage tumors correctly, and may demonstrate the presence and extension of recurrences during follow-up.^16^^,^^28^

All patients underwent surgery, which is the most effective therapy for angiofibromas. There is no consensus on which surgical procedure is best; the most frequently used approach today is the transmaxillary route, which allows better exposure of the tumor, lower morbidity, and no facial scars.^29^

The cure rate, according to the follow-up time of these patients, was 93.75%, which was similar to other published results.^15^^,^^26^

Some studies have reported that better control of surgical bleeding has been associated with improved support for surgery, such as embolization done 24 to 48 hours preoperatively, updated surgical techniques, and professional experience. Embolization may significantly decrease intraoperative bleeding by reducing tumor size, thereby facilitating its removal. As a result, there are low recurrence rates following preoperative embolization. Embolization of the maxillary artery is a relatively safe invasive procedure; a complication of this procedure is embolism into the intracranial circulation, but this event is rare.^15^, ^16^, ^17^, ^18^, ^19^, ^20^, ^21^, ^22^^,^^30^ In the present study, 62.5% of patients underwent embolization without complications; only 2 patients (12.5%) were not embolized, and required intraoperative blood transfusions. These results are similar to other published results.^15^, ^16^, ^17^, ^18^, ^19^, ^20^, ^21^, ^22^^,^^30^

Although the recurrence rate is lower in patients that were embolized,^20^^,^^21^^,^^30^ a few studies have shown opposite results, suggesting that embolization could make it more difficult to identify the full extension of surgical margins, as the tumor becomes smaller; this would increase the recurrence rate.^31^^,^^32^ Our findings were different, since 57% of the 7 patients in which the tumor recurred had not undergone embolization; this is similar to published results, which range from 0% to 57%.^33^ These findings show that decreased bleeding during surgery considerably facilitates tumor removal, decreases the need for intraoperative transfusions, and reduces the recurrence rate in patients undergoing preoperative embolization, as was the case in our sample.^18^^,^^20^^,^^21^

## CONCLUSION

The recurrence rate was associated with the absence of preoperative embolization and advanced tumor stage at the time of diagnosis.

Preoperative selective arterial embolization was the best treatment for angiofibromas among the cases we reviewed; the cure rate was about 94%.

All patients diagnosed with angiofibroma should be investigated with imaging methods, such as computed tomography, and preoperative embolization, to improve surgical planning and reduce intraoperative bleeding.

Follow-up using computed tomography makes it possible to establish the presence and extension of tumor recurrences, or the absence of tumors.
